# Incidence of Carpal Tunnel Syndrome in Distal Radius Fractures Treated by Various Modalities in a Tertiary Care Center: A Single Center Study

**DOI:** 10.7759/cureus.35346

**Published:** 2023-02-23

**Authors:** Jagadish U, Prabhu Ethiraj, Umesh M, Arun H S

**Affiliations:** 1 Department of Orthopedics, Sri Devaraj Urs Medical College, Sri Devaraj Urs Academy of Higher Education and Research, Kolar, IND

**Keywords:** wrist fractures, incidence, median nerve, various modalities, drfs, distal radius fractures, cts, carpal tunnel syndrome

## Abstract

Background

Distal end radius fractures (DRF), which account for 17.5% of all fractures, are the most frequent fracture seen in emergency rooms. In patients with DRFs, delayed carpal tunnel syndrome (CTS) occurs in about 20% of cases. When patients are treated with DRFs using different modalities, CTS results in poor functional outcomes. Our study aims to identify the prevalence of CTS in DRF patients receiving treatment with various modalities.

Materials and methods

Two hundred twenty patients with a history of DRFs who were treated by a variety of modalities at R.L. Jalappa Hospital and Research Center between January 2013 and January 2018 are included in this retrospective analysis. The medical records from the department of the hospital's paperwork were used to gather the patient's information and radiographs. The information was gathered, tabulated, and examined.

Results

In our study, the incidence of CTS in DRF was calculated using a sample size of 220 and found to be 32.73%. The incidence of CTS was shown to be higher in groups with more comminution than less comminution when treatment modalities were analyzed. These groups included closed reduction and internal fixation (CRIF)/open reduction and internal fixation (ORIF) with K wire, external fixation, conservative with the cast, ORIF with variable angle volar locking plate (VAVLP), and ORIF with volar T locking plates (VTLP).

Conclusions

After DRFs, carpal tunnel syndrome is the most significant consequence limiting functional results, hence preventing it requires considerably more attention and care.

## Introduction

The most frequent fracture seen in the emergency room is a distal radius fracture (DRF), which occurs more than 640,000 times a year on average [1]. Distal radius fractures had a bimodal distribution, with one peak mostly composed of young male patients who suffered high-energy fractures and another peak primarily composed of elderly female patients who experienced low-energy trivial fractures [2]. Following the publication of Abbott and Saunders' review article in 1933, it was realized that the correlation between DRF and CTS was a more widespread phenomenon [3].

Distal radius fractures frequently result in arthrosis, malunion, nonunion, tendon rupture, chronic regional pain syndrome (CRPS), ulnar impaction, loss of rotation, finger stiffness, and compartment syndrome. Median nerve compression at the level of the wrist joint, often known as CTS, is another recognized side effect of distal radius fractures [03]. The likelihood of these problems relies on the patient's age, the mechanism of the injury, the kind of fracture, the treatment method, the degree of reduction attained, the posture, and the length of immobilization [4,5].

Due to this, even if there are dangers involved, many surgeons advise a preventive carpal tunnel release at the time of fracture repair [6]. Common symptoms of median nerve compression associated with CTS include diminished feeling, numbness, and pain in its region [7]. Our study's objectives are to ascertain the prevalence of CTS in DRF patients undergoing therapy with various modalities and to analyze the factors affecting CTS incidence in each modality.

## Materials and methods

This study is a retrospective analysis of every distal end radius fracture who visited the Emergency/Outpatient Unit of the Department of Orthopedics at R.L. Jalappa Hospital between January 2013 and January 2018 for a period of five years treated with various available modalities. The sample size is 220. We used the keywords “distal radius fracture,” “open reduction and internal fixation of radius fracture,” “carpal tunnel release,” and “neurolysis” to search the electronic database. Additionally, we examined all surgical documents, radiographs, and medical notes (both from the hospital and the outpatient clinic). The Institutional Ethics Committee of Sri Devaraj Urs Medical College gave approval to this study (approval number DMC/KLR/IEC/56/2022-23).

In our study, patients of either sex over the age of 20 with closed or open, intra or extra-articular distal end radius fractures treated surgically or conservatively were included. Excluded were those related to neurovascular injuries like direct trauma to the median nerve at the time of the accident, possible damage to the median nerve by displaced fracture fragments, carpal tunnel syndrome prior to distal end radius fracture, those fractures handled by neighborhood bone setters, tendon injuries, mono-neuropathies, peripheral non-obstructive neuropathies, polyneuropathies, forearm compartment syndrome.

Orthopedic team members assessed patients in the specialized out-patient clinic after a period of time ranging from six months to one year. Thenar wasting, wrist pain, and carpal tunnel syndrome symptoms including Phalen's test and Tinel sign were also noticed. Serial radiographs were used to assess the fracture's union.

We kept track of all patients who reported hand numbness. The Phalen's test, the Reverse Phalen's test, and the Median nerve compression test were used to analyze each patient's affected and unaffected side wrists clinically, and the results were tabulated. The information was gathered, tabulated, and examined. Collected data were recorded in MS Excel 2016. Data analysis was done using SPSS software version 26.

## Results

One hundred and twenty-six women and 94 men made up the 220 patients in this study. Each of these patients had a different pattern of DRF, such as extra-articular (107) or intra-articular (96) DRFs, volar (14) or dorsal (2) Barton fractures, and Smiths (1) fractures.

In the study, 126 participants were male, and 94 participants were female. The majority 74 (34%) of study participants were aged between 21 and 30 years. A total of 36 (16%) patients belonged to the 41-50 years of age group and 62 (28%) belonged to >50 years of age group. Forty eight (22%) of the participants belonged to the 31-40 years of age group as mentioned in Table 1.

**Table 1:** Age-wise distribution of study participants (n=220)

Age group	Frequency (%)
21-30 years	74(34)
31-40 years	48(22)
41-50 years	36(16)
> 50 years	62(28)

One hundred and ninety fractures were of closed type and 30 were open as mentioned in Table 2. These fractures were treated using a variety of modalities, including internal fixation using Kirschner wires, variable angled volar locking plates (VAVLP), or volar T locking plates, as well as conservative cast placement, closed or open reduction, and these techniques (VTLP). Clinical examination was used to determine the patient's functional status and the condition of the median nerve in the carpal tunnel.

Among the 42 patients who received conservative treatment, 17 (40.47%) patients had CTS. Based on clinical examination, this diagnosis was made in the external fixator group (10 [30.3%]) of 33 patients, the CRIF/ORIF with K-wire fixation group (9 [14.5%] of 62 patients, the ORIF with VAVLP group (17 [40.48%]) of 42 patients, and the ORIF with VTLP group (19 [46.34%]) of 41 patients (Table 2).

**Table 2:** Number of patients treated by each modality, fracture pattern and type. * Conservative method was opted for closed extra-articular fractures. ** External fixation was opted for open intra-articular fractures. † CRIF/ORIF with K wire was most preferred treatment. § ORIF with VAVLP / VTLP was opted in closed intraarticular fractures.

Modality of Treatment	Number of Patients	Fracture Pattern		Type of Fracture	
		Extra-Articular	Intra-Articular	Closed	Open
^* ^Conservative with Cast	42	40	02	41	01
** External Fixation	33	08	25	11	22
^†^CRIF/ORIF with K Wire	62	41	21	58	04
^§^ORIF with VAVLP	42	04	38	40	02
^§^ ORIF with VTLP	41	14	27	40	01

In all, 72 (32.73%) out of 220 patients had a CTS diagnosis. Following data analysis, it was discovered that, as noted in Table 3, 33 (30.84%) of 107 extra articular fractures and 39 (34.51%) of 113 intra articular fractures experienced delayed CTS.

**Table 3:** Number of patients with symptoms and positive clinical tests. * Incidence of CTS is more in conservative group, ORIF with VAVLP/VTLP. ** CRIF/ORIF with K wire has least incidence of CTS.

Modality of Treatment	CTS Symptomatic Patients	Clinical Tests		
		Phalen’s Test	Reverse Phalen’s Test	Median Nerve Compression
^* ^Conservative with Cast	16	14	20	16
External Fixation	09	09	10	10
^**^ CRIF/ORIF with K Wire	10	12	15	09
^* ^ORIF with VAVLP	08	10	12	10
^* ^ORIF with VTLP	13	13	15	14
Total	56	58	69	55

**Figure 1:**
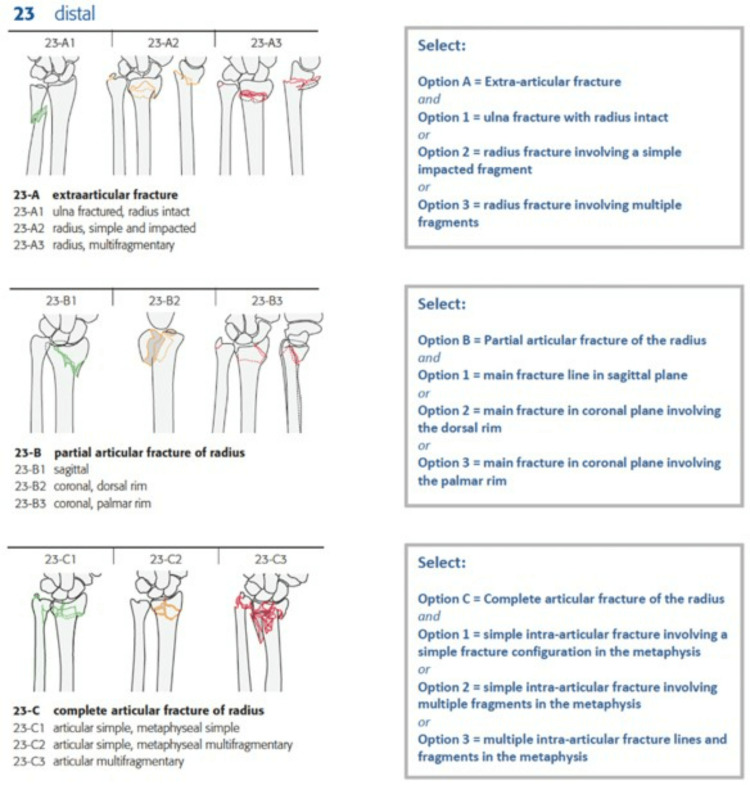
AO classification of wrist fractures AO-Arbeitsgemeinschaft für Osteosynthesefragen

AO fracture classification is mentioned in Figure 1.

**Figure 2:**
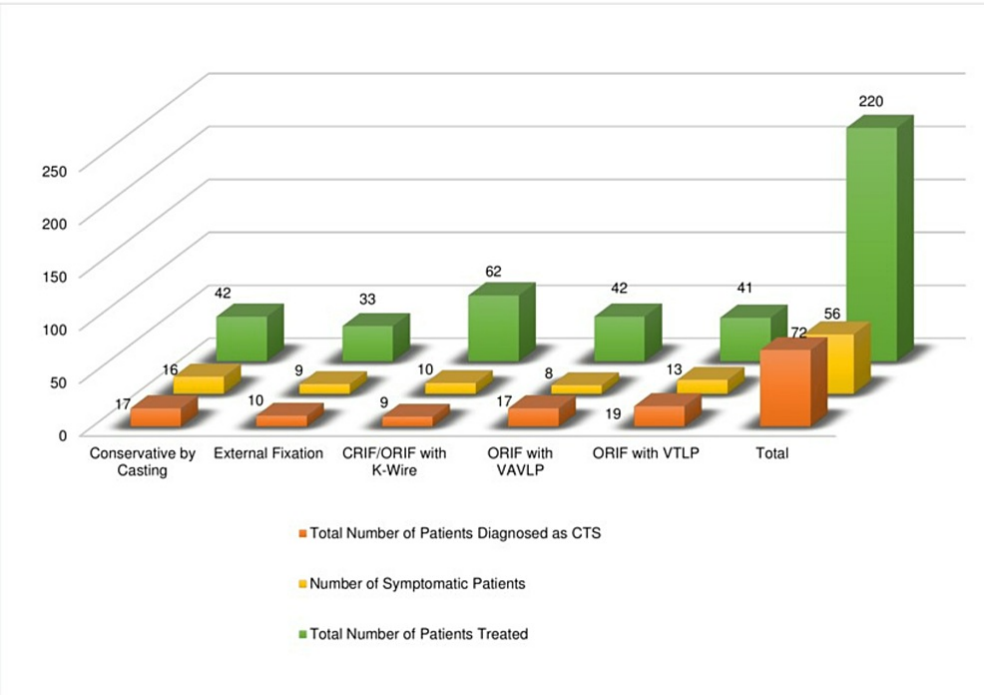
Patients treated with various modalities and became symptomatic/asymptomatic

Results demonstrate a CTS incidence of 30.84% in Group A, 17.85% in Group B, and 40% in Group C when focused on the pattern of fracture and comminution. It also demonstrates that, as was noted, the likelihood of developing CTS rises with increased comminution (Table 4, Figure 2).

**Table 4:** Number of patients in AO classification group, modality of treatment, outcome. * AO Group A fractures were treated by Conservative or K wire fixation have less incidence compared to Group C and incidence increases with increasing comminution. ** AO Group B having less comminuted fractures have least incidence of CTS.

AO Class Group	Number of Patients	Modality of Treatment (CTS)					Total Number Affected
		CONS	External Fixator	CRIF/ORIF with K Wire	ORIF with VAVLP	ORIF with VTP	
^*^A	107	40(17)	8(2)	41(7)	4(2)	14(5)	33 (30.84%)
^**^B	28	2(0)	3(1)	20(2)	2(1)	1(1)	5 (17.85%)
^*^C	85	-	22(7)	1(0)	36(14)	26(13)	34 (40%)
Total	220	42(17)	33(10)	62(9)	42(17)	41(19)	72 (32.72%)

In all, 47 (21.36%) and 25 (11.36%) out of 72 patients developed acute presentation and chronic presentation of carpal tunnel syndrome following distal end of radius fractures treated by various modalities as mentioned in Table 5.

**Table 5:** Number of patients who developed acute/chronic presentations of Carpal tunnel syndrome following various modalities of distal end radius fractures

Modality of treatment	Number of diagnosed Carpal tunnel syndrome patients	Acute presentation	Chronic presentation
Conservative with cast	17	12	5
External fixation	10	2	8
CRIF/ORIF with K-wire fixation	9	2	7
ORIF with VAVLP	17	15	2
ORIF with VTLP	19	16	3
Total	72	47	25

## Discussion

According to the National Hospital Ambulatory Medical Care Survey (NHAMCS) database, hand and wrist fractures accounted for 1.5% of all visits to emergency rooms [8]. Conservative management by closed reduction and casting was and is the most common modality of treatment used for DRFs but with advancement in understanding the mechanisms involved and fixation techniques for DRFs surgical mode is being used in recent days. One of the most prevalent side effects of distal radius fractures is carpal tunnel syndrome, which is also the most common peripheral compressive neuropathy of the upper extremity [9,10]. The onset time of symptoms of CTS as a complication of DRF varies widely from few hours to 24 years, which suggests the existence of wide possible patho-mechanisms [10].

Any factor that alters the carpal tunnel's walls has the potential to cause nerve compression and CTS. Numerous research have proposed patho-mechanisms for the emergence of CTS in DRF, including [5,11]

1. Compression of the median nerve by volarly displaced fragments [12].

2. Injection of local anesthetic during manual reduction leads to an increase in carpal tunnel pressure.

3. Excessive cotton ladder position in the cast.

4. DRF leads to swelling of the carpal tunnel structures.

5. Fracture hematoma causing increased carpal tunnel pressure.

6. The osteosynthesis plate in the volar surface of the distal radius.

7. Excessive manipulation and soft tissue dissection during fixation lead to fibrosis and contractures.

8. Exuberant callus formation during fracture union causing nerve compression.

9. Malunion of fracture leading to malpositioning of the median nerve.

10. Tenosynovitis of flexor tendons and their thickening and tightening during activity.

Repeated or consistent nerve compression and traction may lead to disturbance in intraneural microcirculation, cause lesions at myelin sheath and axonal levels and deform supporting connective tissues [10]. CTS is the most common kind of compressive syndrome and results from median nerve compression at the wrist level [9,10]. History of Flick sign and clinical examination using Tinel’s sign, Phalen’s test (PT), Reverse Phalen’s test, and Median Nerve Compression test (MNCT) is important for diagnosis as mentioned in Table 5.

**Table 6:** Sensitivity and specificity of various clinical tests in diagnosing CTS. * Flick sign is the most sensitive and specific clinical evidence of CTS [9,10]. § Thenar Atrophy is the most specific clinical finding of CTS [9,10]. ** MNCT is the most specific test for CTS [9,10].

Tests	Sensitivity	Specificity
*Flick sign	93%	96%
Phalen’s Test	57 - 68 %	58-73%
**MNCT	64%	83%
Positive Phalen’s Test and MNCT	80%	92%
Tinel’s sign	36-50%	77%
^§^Thenar Atrophy	12-16%	90-94%

Acute CTS must be identified and treated quickly; otherwise, permanent median nerve damage results. Many people with transitory CTS following DRF don't need to have their carpal tunnel surgically released [13]. Before evaluating and treating people with delayed CTS, all possible causes of nerve compression (fibula fragments, hardware, synovitis, systemic causes) should be taken into account. There is an ongoing discussion on the indication and value of preventive CTR in the absence [14].

In our study, 72 patients with DRFs (or 32.73% of the 220 patients with DRFs) had CTS diagnosed. We discovered that the incidence of CTS as a complication of DRFs depends on numerous fundamental parameters, some of which are avoidable and others which are not.

The functional result is significantly influenced by the fracture pattern [6]. When all treatment modalities were taken into account in our study, CTS was determined to be present in 30.84% of 107 AO Group A fractures, 17.85% of 28 Group B fractures, and 40% of 85 Group C fractures. The likelihood of CTS occurring in each group increased as comminution increased. The least amount of volar comminution and least amount of CTS are found in Group B fracture patterns in our study. Eighteen patients with moderate to severe CTS, including those in the highly comminuted groups A3.1-3, B3.3, and C3.2-3, are among the 35 patients. These elements in the findings imply a strong correlation between fracture patterns and the pathophysiology of CTS.

In our investigation, the mode of treatment also seems to play a significant role in the pathogenesis of CTS. When we compared the outcomes of each modality, we discovered that the incidence of CTS was lowest in patients treated with CRIF/ORIF with K wire, at 14.5%, and was significantly higher in those treated with conservative casting (40.47%), ORIF with VAVLP (40.48%), and ORIF with VTLP (46.34%).

As evidenced by the results above, conservative and volar plating techniques have a higher likelihood of developing CTS following DRFs than K-wire fixation and external fixation. Long-term casting with the wrist in palmar flexion may be the cause of the high correlation with CTS in conservative (Cotton Ladder position). Volarly placed fixation plate material and soft tissue dissection that results in fibrosis and contracture are both potential pathogenic causes in volar plating. Both of these circumstances cause the median nerve to pass through the carpal tunnel significantly less, compressing it. Since the aforementioned causes have been present for a long time, these conditions will be linked to symptoms of progressive median nerve compression. It is crucial to understand the pathophysiology generating CTS as a complication in DRFs because all of these factors are avoidable [15]. According to a study by Cooney et al. [16], out of 565 fractures, 177 (31%) had sequelae such as malposition-malunion, radiocarpal or radio-ulnar arthrosis, or persisting neuropathies of the median, ulnar, or radial nerves (45 instances) (30 cases) as mentioned in Table 6.

**Table 7:** Affected patients in other study

Name of the study	Sample size	Affected
Cooney et al. [16].	565	177(31%)
Our study	220	72(32.73%)

A distal radius fracture can cause acute CTS, which appears hours to days later and is thought to be caused by sharply raised compartment pressure in the carpal tunnel. The hematoma formation, fracture displacement, wrist immobilization, and/or soft tissue swelling are likely secondary causes of the critically raised compartment pressure in the context of a DRF. Acute CTS following DRF has been linked to high-energy trauma, repeated closed reduction attempts, fracture displacement, fracture comminution, radiocarpal dislocations, polytrauma, and women under the age of 48.

A change in the structure of the carpal tunnel after the fracture heals is thought to be the cause of delayed CTS, which appears weeks after DRF. The chronically inflamed tenosynovium, volar callus formation, scar formation, offending hardware, and fracture malunion are among the pathophysiologic factors that have been hypothesized for delayed CTS after a DRF [17].

The most significant factor affecting the functional outcome of distal radius fractures is carpal tunnel syndrome. This must be taken into account as one of the most crucial elements during therapy, and measures must be taken to avoid it. The incidence can be reduced by avoiding long-term casting, obtaining almost anatomical reduction, stable fixation, and little manipulation of fractures during reduction, caring for soft tissues during fixation and receiving the proper post-fixation physiotherapy.

Limitations

The following are the study's limitations. This study is neither prospective, randomized, nor controlled. Different surgeons carried out the procedure and the postoperative evaluation, and the follow-up period was not standardized. The primary basis for outcome measurement is patient symptomatology and clinical tests; there are no details regarding confirmatory tests performed like ultrasonography or MRI available. For improved statistical analysis, further prospective study on this topic is required.

## Conclusions

The purpose of this study was to assess the prevalence of carpal tunnel syndrome, a complication of distal radius fractures by clinical methods, as well as the risk factors for its development. In our study, most of the patients who underwent conservative treatment with the cast, ORIF with VAVLP, and ORIF with VTLP developed higher incidences than a patient who underwent ORIF/CRIF with K-wire fixation. In our study, an association of fracture configuration in relation to the incidence of Carpal tunnel syndrome is C>A>B. The main consequence of distal radius fractures that limits functional prognosis calls for considerably higher priority and caution to avoid. To prevent irreversible problems, early detection, and treatment are essential.
